# Did conversion to thoracotomy during thoracoscopic lobectomy increase post‐operative complications and prejudice survival? Results of best evidence topic analysis

**DOI:** 10.1111/1759-7714.14525

**Published:** 2022-07-04

**Authors:** Alfonso Fiorelli, Stefano Forte, Mario Santini, René Horsleben Petersen, Wentao Fang

**Affiliations:** ^1^ Department of Translation Medicine, Thoracic Surgery Unit Università della Campania “Luigi Vanvitelli” Naples Italy; ^2^ Istituto Oncologico del Mediterraneo (IOM) Catania Italy; ^3^ Department of Cardiothoracic Surgery Copenhagen University Hospital Copenhagen Denmark; ^4^ Department of Thoracic Surgery, Shanghai Chest Hospital Jiao Tong University Medical School Shanghai China

**Keywords:** converted thoracoscopic, upfront surgery, video‐assisted thoracoscopic surgery

## Abstract

The potential complications related to unplanned conversion to thoracotomy remains a major concern in thoracoscopic lobectomy and may limit the wide adoption of this strategy. We reviewed the literature from 1990 until February 2022, analyzing all papers comparing successful thoracoscopic lobectomy versus converted thoracoscopic lobectomy and/or upfront thoracotomy lobectomy to establish whether unplanned conversion negatively affected outcomes. Thirteen studies provided the most applicable evidence to evaluate this issue. Conversion to thoracotomy was reported to occur in up to 23% of cases (range, 5%–16%). Vascular injury, calcified lymph nodes, and dense adhesions were the most common reasons for conversion. Converted thoracoscopic lobectomy compared to successful thoracoscopic lobectomy was associated with longer operative time and hospital stay in all studies, with higher postoperative complication rates in seven studies, and with higher perioperative mortality rates in four studies. No significant differences were found between converted thoracoscopic lobectomy and upfront thoracotomy lobectomy. Five studies evaluated long‐term survival, and in all papers conversion did not prejudice survival. Surgeons should not fear unplanned conversion during thoracoscopic lobectomy, but to avoid unexpected conversion that may negatively impact surgical outcome, a careful selection of patients is recommended–especially for frail patients.

## Graphical abstract

Thoracoscopic lobectomy is the treatment of choice for early lung cancer. The fear of conversion should not limit the wide adoption of this procedure, but a careful selection of patients remains mandatory to avoid unexpected conversion that may negatively impact on surgical outcome especially for frail patients.

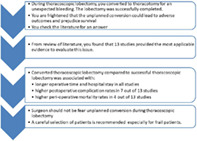

## CLINICAL SCENARIO

A 57‐year‐old man was transferred to our unit for management of lung adenocarcinoma of the left upper lobe. The patient's medical history was unremarkable, and all standard cardio‐pulmonary tests did not contraindicate surgical resection. The tumor was small (15 mm in size) and peripheral and no sign of pleural adhesions and/or of calcified lymph nodes (LNs) were seen on chest computed tomography (CT) scan. No other pathologic lesions were found on whole body fluorodeoxyglucose (FDG)‐positron emission tomography (PET)/CT scans (cT1bN0M0). Based on the current guidelines for the treatment of lung cancer,^1^, ^2^, ^3^ the patient was scheduled for video‐assisted thoracoscopic surgery lobectomy (VATSL).

A standard triportal VATS with anterior access was performed. During the mechanical resection of upper pulmonary vein, the stapler injured the main pulmonary artery resulting in unexpected intraoperative bleeding. Pressure was readily applied with a sponge at the site of bleeding site for an average of 5 minutes, but hemostasis was not achieved. Therefore, an emergent thoracotomy was performed by extending the anterior utility incision for 10–15 cm in length. The main pulmonary artery was proximally closed with a vascular clamp and the defect repaired by angiorraphy using 4–0 polypropylene suture. The blood loss was 550 mL. During arterial clamping, anticoagulation therapy was administered. The planned upper lobectomy with extended lymph node resection was carried out in a standard manner. Chest drainage was left in pleural cavity through the camera incision. Patient was extubated in operating room and then taken to the intensive care unit (ICU).

At this time, were you frightened that the unplanned conversion could lead to adverse outcomes and check the literature for an answer.

## WHY IS THIS QUESTION IMPORTANT?

Lobectomy remains the standard of care for resectable lung cancer and the most authoritative guidelines^1^, ^2^, ^3^ recommended to perform lobectomy by VATS approach especially for early stage lung cancer. VATSL presents real advantages over thoracotomy, including decreased postoperative pain, shorter length of hospital stay (LHOS), less postoperative complications, similar oncological results, and no additional health care costs.^4^, ^5^, ^6^ The most recent analysis of the Society of Thoracic Surgeon (STS) show that 77.7% of lobectomies in 2018 were performed by minimally invasive procedures,^7^ whereas in Europe the rate of VATSL performed in the period 2014–2020 was 49.6%.^8^, ^9^ VATSL remains a complex procedure that requires a demanding learning curve.^10^ Unplanned intraoperative conversion to thoracotomy may affect surgical outcomes, resulting in potential medical problems for the physicians. These concerns may explain the different world‐wide adoption of VATSL.

Although there have been enough evidence suggesting that patients who have received a successful VATSL may benefit from the procedure, it might be at the price of those who have to be converted and suffered from a worse outcome compared to an upfront open thoracotomy. The major purpose of this study was to show that patients undergoing VATSL conversion would have non‐inferior outcomes compared to those who have upfront open lobectomy.

## SEARCH STRATEGY

The study design was structured according to the Preferred Reporting Items for Systematic Reviews and Meta‐Analyses (PRISMA) protocol. A literature review was performed using MEDLINE, PubMed, Scopus, Google Scholar, and Cochrane databases from 1990 until the end of February 2022 to find all studies comparing successful VATS versus converted VATS lung resection and/or upfront thoracotomy lung resection. The following MeSH search headings were used: [vats lung resection.mp. OR VATS LUNG RESECTION] AND [thoracotomy.mp. OR THORACOTOMY/] AND [converted vats lung resection.mp. OR CONVERTED VATS LUNG RESECTION/]. Additional papers, abstracts, chapters of books, letters, and editorials were retrieved from bibliographies by manual research. The Science Citation Index was used to cross reference for further studies that met the criteria of the study.

## SELECTION PROCESS

Papers were included in this review if they fit the following criteria: (i) papers published in the English language; (ii) study population including patients undergoing planned VATS anatomic lung resections that were then converted to thoracotomy; and (iii) results comparing postoperative morbidity and mortality between successful VATS, converted VATS and upfront thoracotomy anatomic lung resections. We excluded (i) studies published in non‐English languages; (ii) reviews, meta‐analyses, abstracts, case reports, and case series; (iii) papers from the same groups (in these cases, only the most recent publication was reported to avoid duplication); and (iv) papers reporting the incidence, the causes, and/or the risk factors for the VATS conversion, but not evaluating the effects of conversion on outcomes and/or survival.

First, the titles of papers were inspected to decide whether they were appropriate to the research purpose. Second, the abstracts of the selected papers were evaluated, and those that were not appropriate were excluded. Third, the remaining articles were entirely inspected to decide their inclusion. Disagreements were judged by the three senior reviewers (M.S., R.P. and V.W.F.) after referring to the original articles.

## END‐POINTS

For each selected paper, the following data were extracted: authors, year of publication, and country; level of evidence based on the criteria of Centre for Evidence Based Medicine^11^; type of resection; incidence, cause, and risk factors for conversion; postoperative outcomes, recurrence, and survival. The end‐points of the study were to evaluate: (i) the incidence, reasons, and the risk factors for conversion; (ii) the postoperative morbidity and mortality; and (iii) recurrence and survival associated with converted VATS as comparison to successful VATS and/or upfront thoracotomy anatomic lung resection.

No specific approval was needed for this study by Local Ethical Committees because it did not involve human subjects.

## RESULTS

**FIGURE 1:**
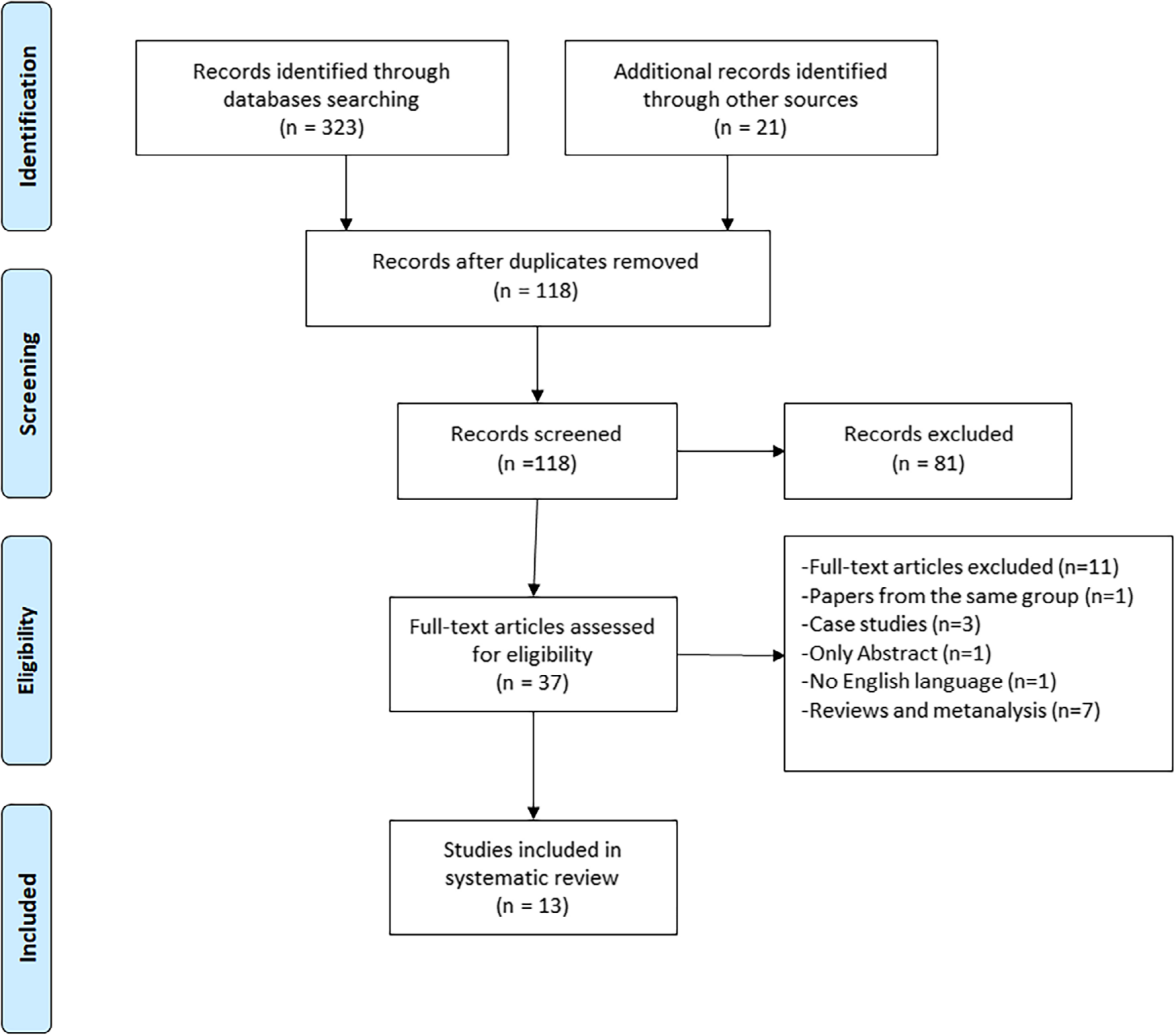
Flow chart of the review according to the PRISMA protocol.

The flow chart of the study is summarized in Figure 1. The initial search using the MeSH heading yielded 323 results and an additional 21 papers were found by manual search from the references of the selected articles; 226 papers were then excluded as duplicate. Among the 118 papers screened, 81 were excluded based on the titles and abstracts. Of the remaining 37 studies, 24 studies were further excluded. Therefore, 13 papers were included in the analysis and summarized in Table 1.^12^, ^13^, ^14^, ^15^, ^16^, ^17^, ^18^, ^19^, ^20^, ^21^, ^22^, ^23^, ^24^

**TABLE 1:** Characteristics of the selected studies

Authors, years, country, level of evidence	Study Groups	Conversions : incidence and reason	Outcomes	Results	Limitations	Conclusions
Servais et al. [12], 2022 United States Retrospective study Level 3a	Successful VATS: 17.399 Converted VATS: 2.148 Study Period: 2015‐2018	Conversion rates: 11% ‐Vascular: 14.3% ‐Anatomy: 68.5% ‐LN: 5.2% ‐Technical: 12% ‐Emergent: 9.6%	Comparison groups	Successful VATS vs. Converted VATS;	Retrospective nature Multiple Centers No upfront thoracotomy group for comparison No analysis of survival and recurrence	Converted VATS was associated with higher mortality and morbidity rates than successful VATS.
			Operation time (min.)	162 vs. 212; *p* < 0.001		
			LOHS (days)	4 vs. 5; *p* < 0.001		
			Perioperative mortality	*p* < 0.001		
			Post‐operative major complication	*p* < 0.001		
			Intra‐operative major complication	*p* < 0.001		
			Post‐operative blood transfusion	*p* < 0.001		
			Risk factors for conversion	‐Age; *p* < 0.0001 ‐BMI; *p* < 0.0001 ‐Male gender; *p* < 0.0001 Hypertension; *p* = 0.0008 ‐Preoperative CT; *p* = 0.0002 ‐Low FEV1; 0.0004 ‐Clinical Stage; *p* < 0.001 ‐Left sided resection; *p* = 0.0002 ‐Positive margin resection; *p* < 0.0001 ‐Lobe location; *p* = 0.01 ‐Low volume center; *p* = 0.0009		
			Comparison	Emergent vs. No‐emergent conversions		
			Mortality	5.5% vs. 1.8%; *p* < 0.001		
Fourdrain et al. [13], 2022 France Retrospective study Level 3b	Successful VATS: 439 Converted VATS: 94 Upfront thoracotomy: 313 Study Period: 2011‐2018	Conversion rates: 17.6% Bleeding: 21 (22%) Oncologic: 6 (6%) Failure of SLV: 13 (13%) Adhesions: 22 (23.%) Technical difficulties: 32 (34%).	Comparison groups	(i) Successful VATS vs. Converted VATS; (ii) Converted VATS vs. Upfront thoracotomy	Retrospective nature Smaller number of patients in the Converted VATS Higher rate of locally advanced tumor stage in upfront surgery group Different type of anatomical resections	Converted VATS and upfront thoracotomy were associated with higher complication rates than successful VATS. Similar survival was found between three study groups.
			Operation time (min.)	(i) 159 vs. 183; *p* < 0.001 (ii) 183 vs. 159; *p* = 0.004		
			Chest tube duration (days)	(i) 3.8 vs.5.9; *p* < 0,001 (ii) 5.9 vs. 6.1; *p* = 0,76		
			LOHS (days)	(i) 6.3 vs. 9.4; *p* = 0.003; (ii) 9.4 vs. 11; *p* = 0.16		
			Postoperative complications	(i) 58 (13%) vs. 21 (22%) *p* = 0.02; (ii) 21 (22%) vs. 88 (28%); *p* = 0.27		
			30‐day mortality	(i) 5 (1%) vs. 2 (2%); *p* = 0.36; (ii) 2(2%) vs. 7 (2%); *p* = 1.0		
			90‐day mortality	(i) 9 (2%) vs. 4 (4%); *p* = 0.26; (ii) 4 (4%) vs. 11 (3%); *p* = 0.76		
			Comparison	Full VATS vs. Converted VATS vs. upfront thoracotomy		
			5‐YSRT before matching			
			Stage I	76% vs. 72% vs. 69%; *p* = 0.47		
			Stage II‐III	77% vs. 40% vs. 53.4%; *p* = 0.016		
			DFSRT before matching			
			Stage I	71 vs. 60% vs. 53%; *p* = 0.013		
			Stage II‐III	63% vs. 35% vs. 41%; *p* = 0.071		
			Comparison	Successful VATS + Converted VATS vs. upfront thoracotomy		
			YSRT after matching	88%, 77% and 65% vs. 92%, 80% and 67% at 1, 3 and 5 years (*p* = 0.22)		
			DFSRT after matching	84%; 64%; and 52% vs. 82%; 67%; and 53% at 1, 3 and 5 years (*p* = 0.49)		
Tong et al.[14], 2020 China Retrospective study Level 3a	Successful VATS: 20.360 Converted VATS: 205 Study period: 2016‐2018	Conversion rate: 1% Bleeding: 29% Adhesions: 28% LN sclerosis: 16% Anatomy: 7.9% Not specified: 5.9% Poor oxygenation: 4% Tumor location: 3.8% R1 resection: 3.8%	Comparison	Successful VATS vs. converted VATS	Retrospective No survival analysis No upfront thoracotomy group for comparison Multiple surgeons Inclusion of sublobar resections	Converted VATS was associated with higher postoperative complication To reduce conversion rate is recommended
			Risk factor for conversion	Age; *p* < 0.001 Male sex; *p* = 0.02 Induction CT; *p* = 0.007 Tumor size; *p* = 0.03 LN involvement; *p* = 0.01; LN calcification; *p* < 0.001 Adhesions; *p* < 0.001 Type of resection; *p* < 0.001 Location of resection; *p* = 0.007 Reoperation; *p* = 0.01 Low surgical experience; *p* < 0.001		
			Operative time (min)	103 vs. 162; *p* < 0.001		
			Blood loss (mL)	95 vs.427; *p* = 0.001		
			Transfusion Intraoperative	0.5% vs. 30%; *p* < 0.001		
			Postoperative	1% vs. 7%; *p* = 0.001		
			Chest drainage (days)	4 vs. 5; *p* < 0.001		
			ICU stay (days)	2 vs. 3; *p* = 0.03		
			LHOS (days)	5 vs. 6; *p* < 0.001		
			Complications ‐Overall	26% vs. 39%; *p* = 0.006		
			‐Pulmonary	26% vs. 37%; *p* = 0.014		
			Readmission to ICU	1% vs. 4%; *p* = 0.03		
			Comparison	Emergent (n=37) vs. non emergent (205)		
			Operative time (min.)	180 vs. 159; *p* = 0.03		
Blood loss (mL)	1% vs. 78%, *p* < 0.001
Sezen et al. [15] 2019 Turkey Retrospective study Level 3b	Successful VATS: 129 Converted VATS: 18 Study period: 2012‐2016	Conversion rates: 12% ‐Bleeding: 6 (33%) ‐Dense adhesions: 7 (38%) ‐Fused fissure: 1 (5.5%) ‐Calcified LN: 4 (22%)	Comparison	Successful VATS vs. Converted VATS	Retrospective series Small sample size Multiple surgeons No difference between emergent and no‐emergent conversion No upfront thoracotomy group for comparison	No significant difference regarding overall postoperative complications and survival between two study groups.
			Operative time (min)	180 vs. 235; *p* = 0.003		
			Blood loss (ml)	263 vs. 562; *p* < 0.001		
			LHOS (days)	4 vs. 5; *p* < 0.001		
			Complication rates			
			‐Overall	20% vs. 22%; *p* = 0.90		
			‐Arrhythmia	3% vs. 16%; *p* = 0.01		
			‐Wound infection	1% vs. 16%; *p* = 0.01		
			5‐YSRT	71% vs. 80%; *p* = 0.54		
			Risk factor for conversion	Age; *p* = 0.015		
Matsuoka et al.[16]; 2019 Japan Retrospective study Level 3b	Successful VATS: 1.527 Converted VATS: 39 Upfront thoracotomy: 89 Study period: 2009‐2014	Conversion rate: 2.5% Tumor extension: 15 (38%) Silicotic LN: 12 (30%) Adhesions: 3 (7%) Poor vision: 3 (7%) Vascular injury: 3 (7%) Bronchial injury: 2 (5%) Stapler misfires: 1 (2%)	Comparison Groups	Successful VATS vs. Converted VATS	Retrospective Study Multiple surgeons No survival analysis No difference between emergent vs. non‐emergent conversion Different type of anatomical resections	VATS converted and upfront surgery were associated with higher complication rates than successful VATS.
			Intraoperative Bleeding (mL)	82 vs. 365; *p* < 0.0001		
			Operation time (min)	121 vs. 187; *p* < 0.0001		
			LHOS (days)	6 vs. 8; *p* = 0.003		
			Complications			
			Grade 2	32% vs. 77%; *p* = 0.005		
			Grade 5	0.4% vs. 5%; *p* = 0.0001		
			Comparison Group	Upfront‐thoracotomy vs. Converted‐VATS		
			Intraoperative Bleeding (mL)	489 vs. 365; *p* = 0.62		
			Operation time (min)	218 vs. 187; *p* = 0.02		
			LHOS (days)	10 vs. 8; *p* = 0.002		
			Complications			
			Grade 2	59% vs. 77%; *p* = 0.48		
			Grade 5	4% vs. 5%; *p* = 0.98		
			Risk factors for conversion	Male; *p* = 0.16 Smoking; *p* = 0.82 Induction therapy *p* = 0.50 Tumor size; *p* = 0.15 Clinical stage; *p* = 0.03		
			Mortality			
			Converted VATS	5%		
			Successful VATS	0.5%		
Upfront thoracotomy	4%
Vallance et al. [17]; 2017 Unite Kingdom Retrospective study Level 3b	Successful VATS: 609 Converted VATS: 75 Study period: 2010‐2015	Conversion rate: 10.9% Vascular: 26; 34% Anatomical: 23;30% Technical: 14; 18% LN: 12; 16.%	LOHS (mean)	6.4 vs. 9.3 *p* < 0.001*	Restrospective study No analysis of recurrence and survival No comparison between emergent vs. non emergent conversion No upfront thoracotomy group for comparison	Converted VATS was associated with more RESPIRATORY failure and 30‐ day mortality as well as longer LOS.
			30‐day mortality	6 (1%) vs. 7 (9%) *p* = 0.003		
			Postoperative complications	224 (36%) vs. 36 (52%); *p* = 0.14		
			Return to theatre	43 (7%) vs. 6 (8%) *p* = 0.78		
			Reoperating for bleeding	6 (1%) vs.2 (2%) *p* = 0.21		
			Readmission within 30 days	41 (6%) vs. 5 (7%) *p* = 0.33		
			Respiratory failure	23 (3%) vs. 10 (14%) *p* < 0.001		
			Empyema	13 (2%) vs. 5 (7%) *p* = 0.023		
			Pneumonia	57 (9%) vs. 12 (17%) *p* = 0.09		
			Arrhythmia	34 (5%) vs. 8 (11%) *p* = 0.10		
			Pulmonary embolus	8 (1%) vs. 2 (2%); *p* = 0.36		
			Myocardial infarction	3 (0.5%) vs. 0; *p* = 0.54		
			Cerebrovascular accident	1 (0.2%) vs. 0; *p* = 0.73		
			PAL	123 (20%) vs. 15 (22%); *p* = 0.96		
Augustin et al. [18]; 2016 Austria Retrospective study Level 3b	Successful VATS lobectomy: 217 Converted VATS lobectomy: 15 Study period: 2009‐ 2012	Conversion rate: 6,5% ‐Vascular injury: 6 (3%) ‐Oncologic: 5 (2%) ‐Technical: 4 (1.7%)	Comparison	Successful VATS vs. Converted VATS	Multiple surgeons No evaluation of emergent conversion No upfront thoracotomy group for comparison No evaluation of survival	Converted VATS was associated with significant longer LOHS
			Chest tube (days)(median)	5 vs. 5; *p* = 0,31		
			Postoperative complications	64 (29%) vs. 5 (33%); *p* = 0.76		
			In‐hospital mortality	2 vs. 0; *p* = 1.0		
			LOHS (days, median)	9 vs. 11; *p* = 0.028		
			Overall survival	*p* = 0.63		
			Recurrence rates	60% vs. 30%; *p* = 0.024		
			Risk factors for conversion	‐Induction treatment; *p* = 0.013 ‐Tumor size; *p* = 0.04		
Byun et al. [19] 2015 Korea Retrospective study Level 3b	Successful VATS: 1.041 Converted VATS: 69 Study period: 2005‐2013	Conversion rate: 6.2% ‐LN: 28 (40.6%) ‐Bleeding: 20 (29%) ‐Oncologic: 11 (15.9%) ‐Adhesions: 5 (7%) ‐Fused fissures: 3 (4%) ‐Failure of single‐lung ventilation: 2 (2.9%)	Comparison	Successful VATS vs. Converted VATS	Retrospective study No analysis of recurrence and survival No comparison between emergent and non‐ emergent conversion No upfront thoracotomy group for comparison	Converted VATS was not associated with increased postoperative morbidity and mortality
			Operation time (min)	150.9 vs. 222; *p* < 0.001		
			Estimated blood loss (mL)	227.5 vs. 692.8; *p* < 0.001		
			Chest tube duration (days)	6.3 vs. 7.1; *p* = 0.14		
			ICU stay (days)	1.4 vs. 3.3; *p* = 0.047		
			In‐hospital stay (days)	8.4 vs. 9.4; *p* = 0.39		
			Complications			
			‐Overall	14 vs. 8; *p* = 0.19		
			‐Respiratory	2 vs. 5; *p* = 0.012		
			‐Non respiratory	12 vs. 3; *p* = 0.76		
			In hospital death	2 vs. 2; *p* = 0.26		
			Risk factor for conversion	‐Age; *p* = 0.031 ‐FEV1; *p* = 0.005 ‐Calcified LN; *p* = 0.02		
Puri et al. [20] 2015 United States Retrospective study Level 3b	Successful VATS: 517 Converted VATS: 87 Upfront thoracotomy: 623 Study period: 2004‐2012	Conversion rate 87 (7%) ‐Vascular injury: 22 (25%) ‐Anatomic reason: 56 (64%) ‐LN: 8 (9%) ‐Technical difficulties or equipment failure: 1 (1%)	Comparison	Successful VATS vs. Converted VATS; Converted VATS vs. Upfront thoracotomy	Retrospective nature Multiple surgeons Upfront thoracotomy and converted VATS group had higher clinical T stage Upfront thoracotomy group presented higher advanced pathologic stage No survival analysis	VATS converted and upfront surgery were associated with higher complication rates than successful VATS. Survival was similar between study groups
			Complication rates	23% vs. 46%; *p* < 0.001; 46% vs. 42%; *p* = 0.56;		
			LHOS (days)	4.6 vs. 7.6; *p* < 0.0001; 7.6 vs. 7.5; *p* > 0.05		
			Transfusion rates	1.3% vs. 16.7%; *p* < 0.001 16.7% vs. 10.3%; *p* > 0.05		
			Surgical mortality	0% vs. 1%; 1% vs. 0.8%; *p* = 0.10		
			Risk factors for conversions	Sex (male) *p* = 0.043		
			Risk factor for long mortality	Age; *p* < 0.0001 Sex (male); *p* = 0.02 Smoking; *p* = 0.019 Low DLCO; *p* = 0.021		
Samson et al. [21] 2013 United States Retrospective study Level 3b	Successful VATS: 148 Converted VATS: 45 Upfront thoracotomy: 91 Study period: 2003‐2009	Conversion rates: 23% ‐ LN calcification: 16 (36%) ‐Adhesions: 15 (33%) ‐Body habitus: 2 (4%) ‐ Other: 2 (4%)	Comparison	Successful VATS vs. Converted VATS	Retrospective series Multiple surgeons No difference between emergent and no‐emergent conversion No evaluation of recurrence and survival	Converted VATS vs. successful VATS was associated with more atrial fibrillation, increased LHOS, longer surgery time, and increase in estimated blood loss. No difference was found between converted and upfront thoracotomy groups Calcified LN was the main predictive factor of conversion
			Operative time (min)	211 vs. 252; *p* < 0.01		
			Blood loss (ml)	150 vs. 325; *p* < 0.01		
			Chest tube	3 vs. 4; *p* < 0.01		
			LHOS (days)	4 vs. 6; *p* < 0.01		
			Complication rates			
			‐Arrhythmia	9% vs. 12%; *p* = 0.04		
			30‐day mortality	1% vs. 9%; *p* = 0.01		
			Risk factor for conversion	LN calcification; *p* = 0.04		
			Comparison	Converted VATS vs. upfront thoracotomy		
			Operative time (min)	252 vs. 215; *p* = 0.02		
			Blood loss (ml)	325 vs. 200; *p* = 0.02		
			Chest tube	4 vs. 3; *p* = 0.02		
			LHOS (days)	6 vs. 5; *p* = 0.07		
			Complication rates			
			‐Arrhythmia	22% vs. 20%; *p* = 0.054		
			30‐day mortality	9% vs. 2%; *p* = 0.10		
Park et al. [22] 2011 Korea Retrospective study Level 3b	Successful VATS: 704 Converted VATS: 34 Study period: 2003‐2008	Conversion rate: 4.6% ‐Silicotic LN: 14 (41%) ‐Vascular or bronchial injury: 11 (32%)) ‐Fused fissure: 4 (11.7%) ‐LN metastasis: 2 (5.8%) ‐Vascular anomalies: 3 (8.8%)	Comparison	Successful VATS vs. Converted VATS	Small sample size Short follow‐up No difference between emergent and non‐emergent conversion No upfront thoracotomy group for comparison	Unexpected conversion to thoracotomy during VATS does not appear to compromise prognosis
			Operating time (minutes)	190 vs. 258; *p* < 0.0001		
			LHOS (days)	7 vs. 10; *p* < 0.0001		
			Operative death	1		
			Complication rates corrected for			
			‐Sex	*p* = 0.45		
			‐Age	*p* = 0.30		
			Survival	*p* = 0.626		
			Recurrence	*p* = 0.767		
Sawada et al. [23] 2009 Japan Retrospective study Level 3b	Successful VATS: 468 Converted VATS: 24 Study period: 2003‐2007	Conversion rate 5% Adenopathies:7 ‐Bleeding: 7 ‐Fused Fissure:4 ‐LN involvement: 1 ‐Others: 5	Comparison	Successful VATS vs. converted VATS	Retrospective No recurrence and survival analysis No comparison between emergent vs. non emergent conversion No upfront thoracotomy group for comparison	VATS is a safe procedure also in case of conversions.
			Operative time (min)	164 vs. 260		
			Blood loss (mL)	144 vs. 420		
			LHOS (days)	10 vs. 12		
			Complications	6% vs. 17%		
Jones et al. [24] 2008 United Kingdom Retrospective study Level 3b	Converted VATS: 26 Upfront thoracotomy: 52 Study period: 1992‐2006	Conversion rate: 10.5% ‐Vascular injury:11 (37%) ‐Extent of disease: 9 (30%) ‐Adhesions: 7 (23%) ‐Stapler misfire: 2 (7%) ‐Contralateral pneumothora x: 1 (3%)	Comparison group	Converted VATS vs. Upfront Thoracotomy	Small sample size No difference between successful vs. converted VATS No difference between emergent and no‐emergent conversion	Converted VATS did not affect surgical outcomes and survival compared to upfront thoracotomy
			Complications			
			‐Overall	13 vs. 25; *p* = 0.093		
			‐Minor	12 vs. 22		
			‐Major	1 vs. 3		
			LHOS (days)	8.3 vs. 9.3; *p* = 0.3		
			5‐YSRT	65% vs. 43%; *p* = 0.1		

**Abbreviations:** CT= computed tomography; DFSR= disease free survival rate; ICU= intensive care unit; LN= lymph node; min= minutes; LOHS= length of hospital stay; PAL= persistent airleaks; VATS= Video‐assisted Thoracoscopic Surgery; SLV=Single lung ventilation; YSRT= year survival rate. [Correction added on 20 July 2022, after first online publication: in table 1, alignment in columns four and five (Outcomes and Results) have been fixed.]

Servais et al.^12^ retrospectively compared the data of successful VATSL (*n* = 17.339) and converted VATSL (*n* = 2.148) for lung cancer. The data was extracted from national database of Society Thoracic Surgery General thoracic Surgery Database (STS GTSD). The overall conversion rate was 11%. Emergent conversion occurred in 9.6% of cases and it was associated with increased mortality compared to non‐emergent conversion (5.5% vs. 1.8%%; *p* < 0.001). Age (*p* < 0.0001), body mass index (BMI) (*p* < 0.0001), male sex (*p* < 0.0001), hypertension (*p* = 0.0008), preoperative chemotherapy (*p* = 0.0002), low FEV1 (*p* = 0.0004), clinical stage (*p* < 0.001), left sided resection (*p* = 0.0002), positive margin resection (*p* < 0.0001), lobe location (*p* = 0.01), and center's experience (*p* < 0.0009) were independent risk factors for conversion. Successful versus converted VATS was associated with a shorter operative time (162 minutes vs. 212 minutes; *p* < 0.001), and LOHS (4 days vs. 5 days; *p* < 0.001). Postoperative mortality (*p* < 0.001), postoperative morbidity (*p* < 0.001), and blood transfusion rates (*p* < 0.001) were higher in converted compared to successful VATS group. The retrospective nature of the study, different centers' experience, the lack of upfront group for comparison, and of survival analysis were all limitations of this study.

Fourdrain et al.^13^ retrospectively compared the data of patients undergoing anatomic resections (segmentectomy, lobectomy, and bilobectomy) for lung cancer by successful VATS (*n* = 439), by converted VATS (*n* = 94) and by upfront thoracotomy (*n* = 313). The conversion rate was 17.6% (*n* = 94) and in 21 cases (22%) it was because of bleeding (emergent conversion).

Operation time (*p* < 0.001), chest tube duration (*p* < 0.001), and LOHS (*p* = 0.003) were shorter in successful VATS than in converted VATS and upfront thoracotomy. Successful VATS was associated with fewer overall complications than converted VATS and upfront thoracotomy (13% vs. 22% vs. 28%, *p* = 0.02, respectively), whereas no significant difference was found between converted VATS and upfront thoracotomy (*p* = 0.27). The conversion did not affect survival. No statistical differences were found in stage‐specific overall survival between the successful VATS, converted VATS, and upfront thoracotomy, with 5‐year overall survival for stage I lung cancer of 76%, 72.3%, and 69.4%, respectively (*p* = 0.47). There was a difference in disease free survival for stage I lung cancer, with 71%, 60.2%, and 53%, respectively at 5 years (*p* = 0.013). There were no statistical differences in early postoperative outcomes, overall survival (*p* = 0.1), or disease free survival (*p* = 0.1) between the emergent and non‐emergent converted patients. The retrospective nature, the small number of patients in the converted VATS, the inclusion of resections different from lobectomy (i.e., sublobar resection and bilobectomy), and the higher rate of locally advanced lung cancer in the upfront thoracotomy group may limit the results.

Tong et al.^14^ retrospectively compared the data of successful VATS (*n* = 20.360) and converted VATS (*n* = 203) lung resections (lobectomy and sublobar resection) for lung cancer. The overall conversion rate was 1%. Emergent conversion occurred in 37 of 203 cases (18%) and it was associated with prolonged operative time (180 minutes vs. 159 minutes; *p* = 0.032), higher blood loss (1.354 mL vs. 232.4 mL; *p* < 0.001), higher rate of intraoperative transfusion (75.7% vs. 1.8%; *p* < 0.001), whereas the differences in overall postoperative complications, pulmonary complications, cardiovascular complications, chest tube duration, ICU stay, and LOHS were not significant. Age (*p* < 0.001), male sex (*p* = 0.02), preoperative chemotherapy (*p* = 0.007), tumor size (*p* = 0.03), LN involvement (*p* = 0.01), LN calcification (*p* < 0.001), pleural adhesions (*p* < 0.001), type of resection (*p* < 0.001), location of resection (*p* = 0.007), ipsilateral reoperation (*p* = 0.01), and surgeon experience (*p* < 0.001) were independent risk factors of conversion. Successful versus converted VATS was associated with a shorter operative time (103 minutes vs. 162 minutes; *p* < 0.001), lower blood loss (95 mL vs. 427 mL; *p* = 0.001), lower intraoperative (0.5% vs. 30%; *p* < 0.001), and postoperative (1% vs. 7%; *p* = 0.001) transfusion rate, shorter chest drainage stay (4 days vs. 5 days; *p* < 0.001), ICU stay (2 days vs. 3 days; *p* = 0.03), and LOHS (5 days vs. 6 days; *p* < 0.001). Postoperative overall complications (26% vs. 39%, *p* = 0.006), pulmonary complications (26% vs. 37%; *p* = 0.014), and readmission to ICU rates (1% vs. 4%; *p* = 0.03) was lower in successful than in converted VATS. The retrospective nature of the study, different surgeons' experience, the inclusion of sublobar resections, the lack of upfront group for comparison, and of survival analysis were all limitations of this study.

Sezen et al.^15^ retrospectively compared successful VATSL (*n* = 129) and converted VATSL (*n* = 18) for lung cancer. Conversion rate was 12% and 6 of 18 (33%) patients underwent emergent conversion for vascular injury. The only significant risk factor for conversion was advanced age (*p* = 0.015). Successful VATS compared to converted VATS was associated with shorter operative time (180 minutes vs. 235 minutes; *p* = 0.003), less intraoperative blood loss (263.9 mL vs. 562.7 mL; *p* = 0.003), and shorter LHOS (4 days vs. 5 days; *p* < 0.001). Despite overall postoperative complications was similar (*p* = 0.90), converted VATS compared to successful VATS was associated with higher rate of arrhythmia (3% vs. 16%; respectively, *p* = 0.01) and wound infection (1% vs. 16%; respectively, *p* = 0.01). No intraoperative and postoperative mortal complications occurred in both groups. The 5 years survival rate in successful and in converted VATS was 74% and 80%, respectively (*p* = 0.54). Yet, no difference was found between emergent and non‐emergent conversion, and there is not the upfront thoracotomy group for comparison.

Matsuoka et al.^16^ retrospectively compared the data of patients undergoing anatomic resections (segmentectomy, lobectomy, and pneumonectomy) for lung cancer by successful VATS (*n* = 1.527), by converted VATS (*n* = 39), and by upfront thoracotomy (*n* = 89). The conversion rate was 17.6% (*n* = 94) and in 3 of 39 cases (7%) it was because of bleeding (emergent conversion). The risk factor for conversion was advanced lung cancer stage (*p* = 0.03). Successful compared to converted VATS was associated with shorter operative time (121 minutes vs. 187 minutes; *p* < 0.0001), and LOHS (6 days vs. 8 days; *p* < 0.003) and with lower intraoperative bleeding (82 mL vs. 365 mL; *p* < 0.0001), lower grade 2 (32% vs. 77%), and lower grade 5 (0.4% vs. 5%; *p* = 0.0001) complication rates. No significant differences were found between converted VATS and upfront thoracotomy. The mortality rate was lower in successful VATS group (0.5%) than in converted VATS (5%) and in upfront thoracotomy group (4%). There were two perioperative deaths in the conversion group because of respiratory complications. The main limitations of this study were the retrospective nature, multiple surgeons who performed operations, the lack of survival analysis between study groups, the lack of comparison between emergent and non‐emergent conversion, and the inclusion of different types of resection from lobectomy (i.e. sublobar resections and pneumonectomy).

Vallance et al.^17^ retrospective compared the data of successful VATSL (*n* = 609) versus converted VATSL (*n* = 75) for lung cancer. The conversion rate was 10.9% and vascular injury was the main reason (34.7%). Converted versus successful VATS was associated with longer LOHS (9 days vs. 6 days, *p* < 0.001) and higher rate of respiratory failure (14.1% vs. 3.8%, *p* < 0.001) and higher rate of 30‐day mortality (9.3% vs. 1%, *p* = 0.003). No recurrence or survival was evaluated. Furthermore, no comparison was found between emergent and non‐emergent conversion, and there was not the upfront thoracotomy group for comparison.

Augustin et al.^18^ retrospectively compared the clinical data of 217 successful VATSL vs. 15 converted VATSL for lung cancer. The conversion rates were 6.5%, because of bleeding (3%), oncologic (5%), and technical (1.7%) reasons. Induction treatment (*p* = 0.013) and tumor size ≥30 mm (*p* = 0.04) were independent risk factors for conversion. Converted versus successful VATS was associated with longer LHOS (11 days vs. 9 days; *p* = 0.028), whereas no significant differences were found regarding overall postoperative complication rate (33.3% vs. 29.5%), median chest drain duration (5 days vs. 5 days) and in‐hospital mortality (0 vs. 1%). More disease recurrences were found in converted vs. successful VATS group (60% vs. 30.5%, *p* = 0.024), but it did not affect the overall survival that was similar (*p* = 0.6). Different surgeons with different skills, the lack of comparison between emergent and non‐emergent conversions, and between converted VATS and upfront thoracotomy were the main limitations of this article.

Byun et al.^19^ retrospectively compared the data of 1.041 successful VATSL and of 69 converted VATSL for lung cancer. Each converted patient was individually matched to three randomly selected non‐converted patients based on date of operation, type of operation, and pathologic stage. The conversion rate was 6.2% because of calcified LN (*n* = 28; 40.6%), vascular injury (*n* = 20; 29%), tumor invasion or extension (*n* = 11; 15.9%), pleural adhesions (5; 7%), fused fissure (*n* = 3; 4%), and failure of single‐lung ventilation (*n* = 2; 2.9%). Converted vs. successful VATS was associated with prolonged operation time (222 minutes vs. 150.9 minutes; *p* < 0.001); higher blood loss (692.8 mL vs. 227.5 mL; *p* < 0.001), and prolonged ICU stay (3.3 days vs. 1.4 days; *p* = 0.047). The differences in overall postoperative complications and in‐hospital deaths were not significant; however, respiratory complications were significantly more common in the converted VATS (*p* = 0.012). There were two deaths in the converted VATSL group because of respiratory complications. Age (*p* = 0.031), FEV1 (*p* = 0.005), and calcified LN (*p* = 0.02) were independent predictive factors for conversion. Converted VATSL was not associated with increased overall surgical morbidity and mortality. The retrospective nature and small sample size were the main limitations of the study. The differences between emergent versus non emergent conversion, and between converted versus upfront thoracotomy, and survival and recurrence rates were not evaluated.

Puri et al.^20^ retrospectively compared the data of successful VATSL (*n* = 517), converted VATSL (*n* = 87), and upfront thoracotomy lobectomy (*n* = 623) performed for lung cancer. The overall conversion rate was 7%. It dropped from 21 of 74 (28%), to 29 of 194 (15%), to 37 of 336 (11%) (*p* < 0.001) over 3‐year intervals. Emergent conversion because of vascular injury occurred in 22 of 87 cases (25%), and it was associated with higher intraoperative blood transfusion compared to non‐emergent conversion (47.4% vs. 4.3%; *p* < 0.001), whereas perioperative morbidity was similar. Male sex (*p* = 0.043) was the only significant prognostic factor for conversion. Successful VATSL was associated with a lower blood transfusion rate (1.3%; *p* < 0.001) and shorter LOHS (4.6 days; *p* < 0.0001) compared to converted VATSL (16.7% and 7.6 days, respectively) and to upfront thoracotomy lobectomy (10.3 days and 7.5 days, respectively), whereas no significant differences were found between converted VATSL and upfront thoracotomy lobectomy. Postoperative complications were more frequent in converted VATS group (46%) than in successful VATS group (23%; *p* < 0.001), but similar to upfront thoracotomy group (42%; *p* = 0.56). No significant difference regarding surgical mortality rate (*p* = 0.10) was found regarding between successful VATS (0%), converted VATS (1%), and upfront thoracotomy (0.8%). Patients undergoing upfront thoracotomy were younger and had a higher incidence of prior lung cancers. Upfront thoracotomy and converted VATS group patients had higher clinical T stage than patients in the VATS group, whereas the upfront thoracotomy group presented higher advanced pathologic stage than other groups.

Samson et al.^21^ retrospectively compared the data of 148 undergoing successful VATSL versus 45 undergoing converted VATSL for lung cancer. Conversion rate was 23% and the main cause of conversion was the presence of LN calcification (33%). Converted VATSL had significantly higher 30‐day mortality (1% vs. 9%; *p* = 0.01), more atrial arrhythmias (9% vs. 12%; *p* = 0.04), increased blood loss (3% vs. 4%; *p* < 0.01), longer operative time (150 minutes vs. 325 minutes; *p* < 0.01), and increased LOHS (4 days vs. 6 days; *p* < 0.01) compared with successful VATSL. On comparison of converted VATSL to upfront open thoracotomy lobectomy, mortality and morbidity rates were similar. Recurrence and survival analysis were not performed. Yet, emergent versus non‐emergent conversions were not compared.

Park et al.^22^ retrospectively compared the data of patients undergoing lobectomy for management of lung cancer (*n* = 603) and benign disease (*n* = 135) by successful VATS (*n* = 704) versus converted VATS (*n* = 34). The conversion rate was 4.6% and the main causes of conversion were the presence of silicotic LN (41%) and bronchovascular injury (32%). Converted compared with successful VATSL was associated with longer operating time (258.8 minutes vs. 190.9 minutes; *p* < 0.0001), LHOS (10.12 days vs. 7.08 days, *p* < 0.0001), whereas complication rates were similar also if corrected for sex (*p* = 0.4579) and age (*p* = 0.307). Survival (*p* = 0.62) and recurrence (*p* = 0.76) rates in patients with lung cancer were not significantly different between the two groups. The main limits of this article were the retrospective nature, the small sample size, the lack of comparison between emergent versus non emergent conversion and between converted VATSL versus upfront thoracotomy lobectomy.

Sawada et al.^23^ retrospectively compared the data of successful VATSL (*n* = 468) versus converted VATSL (*n* = 24) for lung cancer. The conversion rate was 5% and bleeding and adenopathies were the main reasons. Converted VATSL compared to successful VATSL was associated with longer operative time (260 minutes vs.164 minutes), higher amount of bleeding (420 mL vs. 144 mL), higher overall complications (17% vs. 6%), and prolonged LOHS (12 days vs. 10 days). However, there were no life‐threatening perioperative complications or perioperative mortality in both groups. The small sample size, the lack of comparison between emergent versus non emergent conversions, no recurrence and survival analysis, the lack of upfront thoracotomy lobectomy were all limitations of this study.

Jones et al.^24^ retrospectively compared the clinical data of 26 patients undergoing converted VATSL versus 52 patients underwent upfront thoracotomy lobectomy. The converted group was matched 2:1 with upfront thoracotomy group based on age, sex, cancer stage, year, and type of operation. There were no statistically significant differences in postoperative complications between the two groups (*p* = 0.093). There were no in‐hospital deaths in the converted VATSL, but one patient in the control group died of respiratory complication. The survival curve of the converted VATSL seemed to be more favorable than that of the upfront thoracotomy lobectomy, but **s**urvival analysis for cancer‐related death or no‐associated death showed no statistically significant difference (*p* = 0.16). The small sample size and the lack of difference between emergent versus non emergent conversion were the main limits of this article.

## DISCUSSION

Unplanned conversion to thoracotomy remains a major concern in VATSL and it may discourage thoracic surgeons, especially in the early phase of the learning curve, from adopting this approach.^25^ Despite the advantages of VATSL over thoracotomy lobectomy are well defined in literature; there are few and contrasting data regarding the consequences of unplanned converted VATSL on patients' outcome. Therefore, we planned a review study to evaluate whether unplanned converted VATS could increase the postoperative complications and negatively affect survival compared to successful VATSL and/or upfront thoracotomy lobectomy.

### Conversions

In this analysis, conversion to a thoracotomy was reported to occur in up to 23% of cases. Vascular injury, calcified LN, and dense adhesions were the most common reasons for conversion and all studies found a decrease of conversion rate with the increase of the surgeon's experience. Eight studies evaluated risk factors for conversion by multivariable analysis^12^, ^14^, ^15^, ^16^, ^18^, ^19^; the results varied significantly between studies, identifying age, tumor size, BMI, male sex, induction therapy, respiratory disease, history of smoking, side of resection, and surgeon's experience as independent prognostic factors for conversion.

### Postoperative outcomes

In seven studies^12^, ^13^, ^14^, ^16^, ^17^, ^20^, ^21^ converted VATS compared to successful VATS was associated with higher rate of post‐operative complications, whereas six studies showed no significant differences.^15^, ^18^, ^19^, ^22^, ^23^, ^24^ Four of 13 studies found a higher rate of peri‐ and postoperative deaths after conversion because of cardio‐respiratory complications.^12^, ^16^, ^19^, ^21^ Longer operating time, lung manipulation with air‐leaks, increased blood loss, and long‐time ICU stay related to conversion and pre‐operative patients' comorbidities were likely explanations. Because the advantages of VATS compared to thoracotomy are well defined in literature, the comparison group for converted VATS should also include patients undergoing upfront thoracotomy. However, this issue was evaluated in only four studies,^13^, ^16^, ^20^, ^21^ presenting comparable results between converted VATS and upfront thoracotomy. An additional critical point was to distinguish the reasons for conversions because they could have a different impact on surgical outcome. Emergent conversions because of vascular injuries were life‐threatening conditions conversely to non‐emergent conversions performed for technical reasons (i.e., pleural adhesions, limited space, stapler malfunction, difficult to perform single lung ventilation, and calcified nodes). Despite all, only 4 of 13 studies compared emergent versus non‐emergent conversions.^12^, ^13^, ^14^, ^20^ In three studies^13^, ^14^, ^20^ no differences were found, but one study^12^ found that emergent conversion was associated with an increased mortality.

### Survival

Five of 13 studies evaluated the long‐term survival and found no significant difference between successful VATS and converted VATS.^13^, ^15^, ^20^, ^22^, ^24^ One study found a higher recurrence rate in converted compared to successful VATS group.^18^ Emergency conversion could lead to unintended disruption of cancer cells with dissemination of malignancy and higher risk of recurrence. By contrast, converted VATS group and/or upfront thoracotomy group included higher rate of patients with advanced cancer, likely more difficult to successfully resect by VATS. Therefore, it remained difficult to show whether these results were because of the negative impact of conversion, or the intergroup differences.

### Recommendations from the analysis

VATSL remains a safe and feasible procedure. It should be strongly considered for the majority of patients undergoing lobectomy and the fear of unplanned conversion should not limit it being widely adopted. Because converted VATS could be associated with increased rates of post‐operative complications, as found in seven studies^12^, ^13^, ^14^, ^16^, ^17^, ^20^, ^21^ and of peri‐ and postoperative death as observed in four studies,^12^, ^16^, ^19^, ^21^ the appropriate selection of patients remain mandatory to avoid unexpected conversion, especially in frail patients who would be considered high risk for thoracotomy. The preoperative identification of risk factors as calcified lymph node, advanced stage lung cancer, bronchovascular abnormalities, induction chemo‐radiotherapy, and dense adhesions may help surgeons in selecting appropriate patients for VATSL.^26^, ^27^ Furthermore, in case of unexpected complications, surgeons should be ready to convert to thoracotomy because patient safety must remain the primary objective of surgery. Delayed conversion and/or an unsuccessful attempt to manage complications by VATS increase the risk of intraoperative events that may be fatal.

## LIMITATIONS

This article presented several limitations that should be taken in account before drawing definitive conclusions. All studies were retrospective.^12^, ^13^, ^14^, ^15^, ^16^, ^17^, ^18^, ^19^, ^20^, ^21^, ^22^, ^23^, ^24^ Obviously, the intraoperative conversion cannot be predicted and therefore, it makes it impossible to plan prospective randomized studies. Therefore, the different characteristics of the study groups (i.e., tumor stage, and pre‐operative morbidity) could affect the results. Despite all, only 1 of 13 studies used propensity score matching analysis to balance the intergroup differences.^13^ Yet, the type of resection and the outcomes were not standardized between the studies as well as surgeon's experience. Three of 13 studies included not only lobectomy,^13^, ^14^, ^16^ but also sublobar (i.e., wedge resection and segmentectomy) and/or more extended resections (i.e., bilobectomy and pneumonectomy). Only 4 of 13 studies compared emergent versus non‐emergent conversion,^12^, ^13^, ^14^, ^20^ 5 out of 13 studies evaluated long term survival,^13^, ^15^, ^20^, ^22^, ^24^ and 4 of 13 studies included upfront thoracotomy lobectomy for comparison.^13^, ^16^, ^20^, ^21^ Yet, patients undergoing upfront thoracotomy presented higher rates of locally advanced cancer (usually considered difficult to resect by VATS) compared to patients undergoing converted VATS, making challenging any comparisons.

## CONCLUSIONS

VATSL is the treatment of choice for early lung cancer. The fear of conversion should not limit the wide adoption of VATSL, but a careful selection of patients remains mandatory to avoid unexpected conversion that may negatively impact on surgical outcome especially for frail patients. Finally, the conversion should never be considered as a treatment failure. The decision to convert must be made promptly especially in case of life‐threatening intraoperative complications.
